# A novel *O*-methyltransferase Cp4MP-OMT catalyses the final step in the biosynthesis of the volatile 1,4-dimethoxybenzene in pumpkin (*Cucurbita pepo*) flowers

**DOI:** 10.1186/s12870-024-04955-3

**Published:** 2024-04-17

**Authors:** Marion Christine Hoepflinger, Monica Barman, Stefan Dötterl, Raimund Tenhaken

**Affiliations:** 1 Department of Environment & Biodiversity, Paris Lodron University Salzburg, Hellbrunnerstraße 34, Salzburg, 5020 Austria; 2 Leibniz Institute of Vegetable and Ornamental Crops (IGZ), Theodor-Echtermeyer-Weg 1, 14979 Großbeeren, Germany

**Keywords:** *O*-methyltransferase (OMT), *Cucurbita pepo* 4-methoxyphenol-*O*-methyltransferase (Cp4MP-OMT), 1,4-dimethoxybenzene (1,4-DMB), 4-methoxyphenol (4-MP), Flower scent, Styrian oil pumpkin, Insect pollination

## Abstract

**Background:**

Floral scents play a crucial role in attracting insect pollinators. Among the compounds attractive to pollinators is 1,4-dimethoxybenzene (1,4-DMB). It is a significant contributor to the scent profile of plants from various genera, including economically important *Cucurbita* species. Despite its importance, the biosynthetic pathway for the formation of 1,4-DMB was not elucidated so far.

**Results:**

In this study we showed the catalysis of 1,4-DMB in the presence of 4-methoxyphenol (4-MP) by protein extract from Styrian oil pumpkin (*Cucurbita pepo*) flowers. Based on this finding, we identified a novel *O*-methyltransferase gene, *Cp4MP-OMT*, whose expression is highly upregulated in the volatile-producing tissue of pumpkin flowers when compared to vegetative tissues. OMT activity was verified by purified recombinant Cp4MP-OMT, illustrating its ability to catalyse the methylation of 4-MP to 1,4-DMB in the presence of cofactor SAM (*S*-(5′-adenosyl)-*L*-methionine).

**Conclusions:**

Cp4MP-OMT is a novel *O*-methyltransferase from *C. pepo*, responsible for the final step in the biosynthesis of the floral scent compound 1,4-DMB. Considering the significance of 1,4-DMB in attracting insects for pollination and in the further course fruit formation, enhanced understanding of its biosynthetic pathways holds great promise for both ecological insights and advancements in plant breeding initiatives.

**Supplementary Information:**

The online version contains supplementary material available at 10.1186/s12870-024-04955-3.

## Introduction

Floral scents are intricate blends of volatile compounds, primarily consisting of benzenoids and phenylpropanoids, terpenoids, fatty acid derivatives, and nitrogen-containing compounds. The distinct floral fragrance notes generated across flowering plants result from variations in the type and quantity of the volatiles emitted [1, 2]. Many wild plant species and numerous crop plants rely on pollinators for successful fruit formation [3–5], whereby floral scents play a pivotal role in attracting pollinators, ensuring effective pollination [6–8]. Insect pollinators perceive specific volatile compounds with low odor thresholds and showcase the ability to discriminate among compounds. Thus, floral volatiles affect the rate of pollinator visits to flowers and the kinds of pollinators attracted [9, 10]. In the last decades, various compounds attractive to pollinators were described, including benzyl acetate, methyl benzoate, methyl salicylate, 2-phenylethanol, *p*-anisaldehyde, linalool, and geraniol [8, 11, 12]. Also, the biosynthesis of a few of the attractive volatiles has been elucidated, and various enzymes involved in their formation have been characterized [13–16]. However, there remains a gap in understanding the biosynthetic pathways responsible for most of the floral scent compounds that are important for pollinator attraction, and finally for successful pollination [8].

**Fig. 1:**
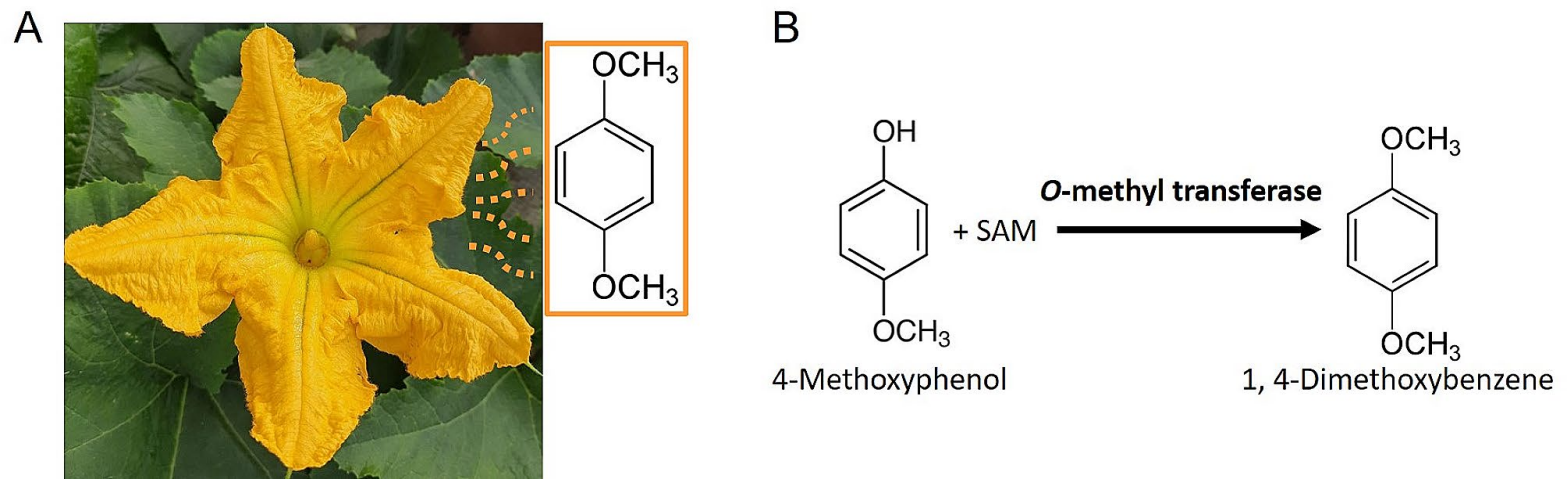
Predicted formation of 1,4-dimethoxybenzene in Styrian oil pumpkin flowers. Pictorial representation showing the emission of 1,4-dimethoxybenzene from a Styrian oil pumpkin flower **(A)** and the proposed enzymatic conversion of 4-methoxyphenol and *S*-(5′-adenosyl)-*L*-methionine (SAM) to 1,4-dimethoxybenzene by an *O*-methyl transferase **(B)**

One such molecule known for eliciting physiological and behavioural responses in a diverse range of insect pollinators is 1,4-dimethoxybenzene (1,4-DMB), a methoxylated aromatic volatile compound [8, 9, 17–19]. It serves as a major floral volatile in plant species of several genera, including *Salix* [17, 20], *Lithophragma* [21], *Nelumbo* [22], *Catasetum* [23], *Allium* [9], and *Fragaria* [24]. Additional, 1,4-DMB is a predominant volatile from the flowers of commercially significant *Cucurbita* species, such as various varieties of *C. pepo* and *C. maxima* (Fig. 1A; [25–30]). Many *Cucurbita* species and closely related cucurbits are monoecious plants, featuring separate male and female flowers [31, 32]. Pollinators are mainly bees that play a crucial role in transferring pollen from male to female flowers [31, 33, 34], and 1,4-DMB is likely involved in attracting them (e.g. *Apis mellifera*, *Bombus terrestris*) to the flowers [18, 30, 35], as was recently shown for a florivorous beetle that also visits *Cucurbita* flowers [30, 36–38]. Despite its pivotal role in pollinator attraction, the enzymes and substrates orchestrating the formation of this volatile compound remain unknown.

Previously, however, researchers have elucidated the ultimate pathway genes and corresponding enzymes involved in the synthesis of structurally similar methoxylated aromatic compounds. Instances include the biosynthesis of 3,5-dimethoxytoluene and 1,3,5-trimethoxybenzene in *Rosa chinensis* flowers [39, 40]. *O*-methyltransferases (OMT) derived from floral petal tissue are involved in the catalysis of these compounds [39, 40]. Similarly, guaiacol OMT was isolated from petal tissue of *Silene* flowers and demonstrated its involvement in the methylation of guaiacol (2-methoxyphenol) to veratrole (1,2-dimethoxybenzene) [41, 42].

In our ongoing investigations on the chemical ecology of pollination in Styrian oil pumpkin (*Cucurbita pepo* subsp. *pepo* var. *styriaca*), substantial emissions of 1,4-DMB were recorded from the flowers (primarily petals) of also this variety of *C. pepo* (Barman et al., unpublished), and here, we studied its biosynthesis. Unravelling the biosynthetic pathway involved in the production of this compound holds promise for both ecological understanding and plant breeding initiatives. We present a novel OMT gene from pumpkins, prominently upregulated in the volatile-producing tissue of flowers. The purified recombinant protein exhibits OMT activity, catalysing the final methylation of 4-methoxyphenol resulting in 1,4-DMB (Fig. 1B).

## Methods

### Chemicals and reagents

Hydroquinone, 4-hydroxybenzoic acid, 4-methoxyphenol (4-MP), *S*-(5′-Adenosyl)-*L*-methionine chloride dihydrochloride (SAM), and acetosyringone were purchased from Sigma-Aldrich (Merck, Darmstadt, Germany). Tris, MgCl_2_, β-mercaptoethanol were obtained from Roth (Karlsruhe, Germany).

### Plant material

Seeds of Styrian oil pumpkin (*Cucurbita pepo* subsp. *pepo* var. *styriaca* Greb.) were obtained from Saatzucht Gleisdorf GmbH Austria. The plants were grown in pots (20 cm height and 25 cm diameter) in the greenhouse of the Botanical Garden of Salzburg University between May and August 2022 in a substrate containing three parts of steamed compost and one part of steamed soil (Einheitserde®, Profi Substrat). The flowers only bloom for a few morning hours on a single day each, between c. 05:00 h and c. 10:00 h.

For transient protein expression, tobacco plants (*Nicotiana benthamiana* Domin.) were grown on standard fertilized soil (type ED73) with a 16/8 h light/dark cycle in a growth chamber (23 °C during light and 22 °C during dark phase; relative humidity was set to 60%). Four-week-old plants were used for *Agrobacterium*-mediated transient protein expression.

### Sequence analyses

To identify putative OMT sequences, transcriptomic datasets of leaf and flower tissues of Styrian oil pumpkin were compared. Total RNA was extracted as described below and sent to Novogene (Cambridge, UK) for sequencing. Datasets obtained from 30 million paired end reads (2 × 150 bp) were used for *de novo* transcriptome assembly with Trinity (GitHub version 2.11.0) using default settings. Assembled transcripts were annotated with Diamond (GitHub version 2.11.0) [43]. We identified a methyltransferase gene, which showed a strong upregulation [3272 FPKM (fragments per kilobase of transcript per million reads mapped)] in flower as compared to leaves (10.45 FPKM) with a sequence that is homologous to known OMTs.

We performed phylogenetic analyses of methyltransferases acting on OH-groups of aromatic ring structures. Sequences, annotated in GenBank as OMTs, were aligned with ClustalX. A tree was constructed with TreeViewer V2.2.0.

For detection of protein families, domains, and regions, InterPro was used [44]. Following sequences were analysed: Cp4MP-OMT (this paper), *Rhaphiolepis* CCOMT (LC127201), *Silene* GMT1 from [42], tomato COMT (NP_001306101), *Arabidopsis* CCOMT (AAL07162), and human COMT (AAA68927).

### RNA and cDNA preparation, and cloning

Pumpkin leaf tissues (tendrils), which do not release 1,4-DMB, and fully bloomed flower petals, that release 1,4-DMB (Barman et al., unpublished) were collected (100 mg fresh weight each) at 7:00 h. Samples were snap-frozen in liquid nitrogen and stored at -80 °C until analysis. Total RNA was extracted with TRI-Reagent according to the supplier’s protocol (Sigma Aldrich). Any residual genomic DNA was digested using RNase-free Dnase (EN0521, Thermo Fischer Scientific, Waltham, MA, USA). First-strand cDNA was synthesized from 2 µg total RNA by M-MuLV Reverse Transcriptase (RevertAid EP0441, Thermo Fischer Scientific) combined with an anchored oligo(d)T primer-mix according to manufacturer’s instructions. The obtained cDNA was used as a template for cloning of *Cucurbita pepo* 4-methoxyphenol-*O*-methyltransferase (*Cp4MP-OMT*) sequence as well as for expression analysis using quantitative PCR (both described below).

To create the vector expressing *Cp4MP-OMT*, we introduced a linker as well as the pumpkin sequence in a single hot fusion reaction into the expression vector (pEAQ-His::GFP [45]) according to the protocol described in [46]. Phusion High-Fidelity DNA polymerase (F530S, ThermoFischer Scientific) was used according to manufacturer’s instructions and the primers HF_GFP_linker_4MP-OMT_F (5′- GCATGGATGAACTATACAAA**GGAGGTGGATCCGGCGGTGGTTCCGGA**ATGGAAGAGAAGGTGGATGA-3′; bold letters indicate the linker sequence) and HF_4MP-OMT_pEAQ_R (5′-TGAAACCAGAGTTAAAGGCCTTAAGGGTACACCTCAATAA-3′). The obtained PCR product was purified using a PCR clean up kit (#K0701, ThermoFischer Scientific). The expression vector (pEAQ-His::GFP) was cut with XhoI (FD0694, ThermoFischer Scientific). 25 ng of this cut pEAQ-His::GFP and 50 ng of Gly/Ser-linker::4MP-OMT were used for the hot fusion reaction. The resulting vector (pEAQ-His::GFP::Gly/Ser-linker::Cp4MP-OMT) was transformed into *E. coli* (strain XL-1) to amplify the plasmid. The expression vector was purified using GeneJET Plasmid Miniprep kit (#K0502, ThermoFischer Scientific) and the sequence was confirmed by sequencing (GenBank accession number for Cp4MP-OMT: OR913727).

### Protein expression in tobacco leaves

The expression vector (pEAQ-His::GFP::Gly/Ser-linker::Cp4MP-OMT) as well as the empty pEAQ-His::GFP vector were transformed into electro-competent *Agrobacterium tumefaciens* (strain GV3101) and plated on YEB plates containing antibiotics (50 µg/ml kanamycin, 25 µg/ml gentamycin). Plates were incubated at 28 °C for 2 days until colony forming units (cfu) were visible. A single cfu was taken from each transformation and spread on a fresh YEB plate containing antibiotics for further growth. From these plates two to three loops of *A. tumefaciens* carrying the expression vector (either with or without *Cp4MP-OMT*) were taken and grown for about 16 h at 28 °C in 10 ml liquid YEB (including antibiotics) to late-exponential phase under continuous shaking (about 200 rpm). The cultures were harvested by centrifugation (6000 x g, 5 min, room temperature). The pellets were resuspended in 5 ml infiltration buffer [10 mM MES/KOH (pH 5.6), 10 mM MgCl_2_, and 150 µM acetosyringone] each and bacterial concentration was determined photometrically (OD_600_). Suspensions were adjusted to OD_600_ = 0.5 with infiltration buffer and incubated for another 2 h at room temperature under continuous shaking. The suspensions were infiltrated into four-weeks-old *N. benthamiana* leaves using a 1 ml syringe. To enable precise recognition of transiently transformed leaf parts, infiltrated leaf areas were marked with a pen. After expression for 5 days in a growth chamber with 16/8 h light/dark cycle (temperature and humidity as described above) expression was confirmed by GFP fluorescence under black light. Marked areas were cut and tested for protein expression either as crude extract or as purified His-tagged Cp4MP-OMT.

### Affinity purification of his-tagged protein, SDS-PAGE, and Western blot analyses

Leaf areas of tobacco transiently expressing *Cp4MP-OMT* were cut and grounded in liquid nitrogen using mortar and pestle. Three ml of LEW buffer (50 mM NaH_2_PO_4_, 300 mM NaCl, pH 8.0 from Protino® Ni-TED kit, Macherey-Nagel, Germany) were added to 1.5 g of fresh leaf material. While thawing on ice the extract was shaken by hand as well as vortexed vigorously. After thawing, the extract was incubated for another 10 min on ice while constantly shaking. This crude extract was used on the one hand for direct measurement of enzymatic activity and on the other hand for protein purification under native conditions. To purify the His-tagged Cp4MP-OMT from the crude leaf extract, Protino® Ni-TED kit was used (Macherey-Nagel, Germany). All purification steps were performed on ice. Cell debris was pelleted by centrifugation (15,000 x g, 10 min, 4 °C). The resulting supernatant was centrifuged again (15,000 x g, 10 min, 4 °C) and filtered through two layers of miracloth (Calbiochem/Merck, Darmstadt, Germany) to remove any residual particles. Protino Ni-TED 1000 pre-packed columns were equilibrated with 4 column volumes of LEW buffer. Supernatant was loaded onto the column and drained by gravity. The column was washed twice with 2 column volumes of LEW buffer. Proteins were eluted using 2 column volumes of elution buffer (50 mM NaH_2_PO_4_, 300 mM NaCl, 250 mM imidazole, pH 8.0), supplemented with 20% glycerol and stored at -20 °C for short-term and at -80 °C for long term storage.

For SDS-PAGE and Western blot analysis either crude extract or samples from purification steps were used. Following separation on a 12.5% acrylamide separation gel, the proteins were either directly stained with Coomassie Brilliant Blue or blotted onto a polyvinylidene difluoride membrane (PVDF; Merck, Darmstadt, Germany) for 70 min at 100 V. The membranes were blocked for 60 min in TBST-BSA (2%, w/v) at room temperature followed by addition of a monoclonal anti-(His)6-tag antibody (DIA-900-200, Dianova, Leipzig, Germany; dilution 1:1000). After incubation for 60 min the PVDF membrane was washed 3 times (5 min each) in TBST. The secondary antibody [monoclonal anti-mouse IgG(HRP), A4416, Sigma-Aldrich; dilution 1:8000] was applied for 60 min in TBST. After another wash step (3 times for 10 min each in TBST) proteins were detected using the chemiluminescent detection kit CheLuminate-HRP PicoDetect Extended (AppliChem, Darmstadt, Germany) according to manufacturer’s instructions. Chemiluminescence was detected by LAS 3000 mini imaging system (Fujifilm, Tokyo, Japan).

### Floral protein extraction and OMT enzyme assays

Petal tissues (fresh weight 200 mg) from fully bloomed flowers were collected at 7:00 h. Samples were snap-frozen in liquid nitrogen and stored at -80 °C until analysis. For protein extraction, the petal tissue was ground to fine power using a ball bead homogenizer (Retsch MM 301). Extraction and assay of OMT enzyme was performed by the method described by [41] with slight modifications. Protein was extracted in extraction buffer (750 µl) containing 100 mM Tris-HCl, pH 7.5, 5 mM MgCl_2_, 10 mM β-mercaptoethanol, 10% glycerol [v/v], and 3% insoluble polyvinylpolypyrrolidone [w/v]. Extraction buffer without 3% insoluble polyvinylpolypyrrolidone is named as buffer A. The floral extract was centrifuged at 4 °C to pellet down the insoluble floral tissue and obtain the soluble protein extract as the supernatant. Further the soluble protein extract was desalted on PD10 columns (GE healthcare) which was equilibrated with extraction buffer without 3% insoluble polyvinylpolypyrrolidone (named as buffer A).

To determine OMT enzyme activity for formation of 1,4-DMB, we used desalted crude protein extract (100 µg) and incubated that with 1 mM of S-(5′-adenosyl)-L-methionine (SAM) and 1 mM of either of three potential substrates [4-hydroxybenzoic acid (4HBA), hydroquinone (1,4-dihydroxybenzene, HYQ), 4-methoxyphenol (4-MP)] in a final volume of 500 µL maintained with buffer A for 30 min at room temperature in microcentrifuge tubes (1.5 ml). The reaction volatile product formed was collected by dynamic headspace and analysed by GC/MS (gas chromatography / mass spectrometry) as described below.

### Collection and analysis of volatile compounds

The volatile compounds were collected by dynamic headspace [17]. Briefly, after the reaction time of 30 min (mentioned in the previous section), the assay mixture (500 µl) was transferred to a Munktell Filtrak™ grade 3 filter paper (diameter: 90 mm). This filter paper, along with the empty reaction tube, was sealed inside a polyethylene oven bag (Toppits®, Minden, Germany; dimensions of 15 cm × 15 cm) for 5 min to accumulate the target volatiles released from the filter paper. Promptly after that, a small cut was made in one corner of the bag through which an adsorbent tube comprising of a quartz glass cylinder (length 25 mm, inner diameter 2 mm) filled with 3 mg mixture (1:1) of Tenax-TA (mesh 60–80, Supelco, Merck KGaA, Darmstadt, Germany) and Carbotrap B (mesh 20–40, Supelco), attached to a membrane pump (G12/01 EB; Rietschle Thomas Inc., Puchheim, Germany), was inserted. The volatiles released from the filter paper were trapped in the adsorbent tube by maintaining a flow rate of 200 ml/min for 5 min.

The samples were analysed using an automated TD (thermal desorption) system (model TD-20, Shimadzu, Japan) coupled to a GC/MS (model QP2010 Ultra EI, Shimadzu, Japan), which was equipped with a ZB-5 fused silica column (5% phenyl polysiloxane; length of 60 m, inner diameter of 0.25 mm and film thickness of 0.25 μm) as described previously in [47, 48]. The identification of the volatile compounds was performed using the libraries available for mass spectral and retention index data (NIST11, Wiley9, Essential oils, FFNSC 2, Adams 2007). Compounds were also authenticated by comparison with standard compounds available in the Plant Ecology lab of Salzburg University. Quantification of 1,4-DMB was done based on a concentration curve obtained from authentic 1,4-DMB.

### Gene expression analyses

For gene expression analysis, quantitative PCR (qPCR) was used. RNA as well as first-strand cDNA were prepared from pumpkin vegetative tissue (tendrils) and fully bloomed flower petals as described above. Quantitative PCR was performed on an AriaMx cycler (Agilent Technologies, California, US) using 2x qPCRBIO SyGreen Mix Lo-ROX (PCRBIOSYSTEMS, London, UK) according to manufacturer’s instructions. Following primers were used: 4MP-OMT (OMT_fwd: 5′-ACAGCTTTGTGGAGGTTGCT-3′, OMT_rev: 5′-GGGCGTGGCTATGGATAACA-3′) and as a housekeeping gene *Cucurbita pepo *Actin (CpACT: CpACT_fwd: 5′-TTCCCGTTCAGCAGTAGTGG-3′, CpACT_rev: 5′-GCCCTCCCTCATGCAATTCT-3′). Means of resulting C_t_ values were used for calculation of *Cp4MP-OMT* expression relative to *CpActin* according to [49].

### Statistical analysis

The statistical tests related to volatile analyses were performed using R programming language (version 4.2.2 [50]) in the Rstudio platform (version 2021.09.2 + 382 “Ghost Orchid” Release). The amounts of 1,4-DMB in the headspace samples were graphically displayed via boxplots using “ggplot2” package (version 3.4.0). In these boxplots, the thick black horizontal lines indicate the median, boxes indicate the interquartile range, whiskers indicate the minimum/maximum range without outliers, and black dots represent outliers. Kruskal-Wallis-test followed by Dunn’s test using “dunn.test” package in R were performed to assess the pairwise comparison of 1,4-DMB formation using different substrates with crude floral protein extract. Mann-Whitney *U* test was performed to assess the difference between the amounts of 1,4-DMB formation by control and recombinant protein. Statistical tests (unpaired t-test) and graphical representation related to gene expression analyses between petal and leaf tissues were performed using GraphPad Prism 9 (version 9.1.2).

## Results

### Protein extract of pumpkin flower petals shows the ability to catalyse 1,4-DMB

**Fig. 2:**
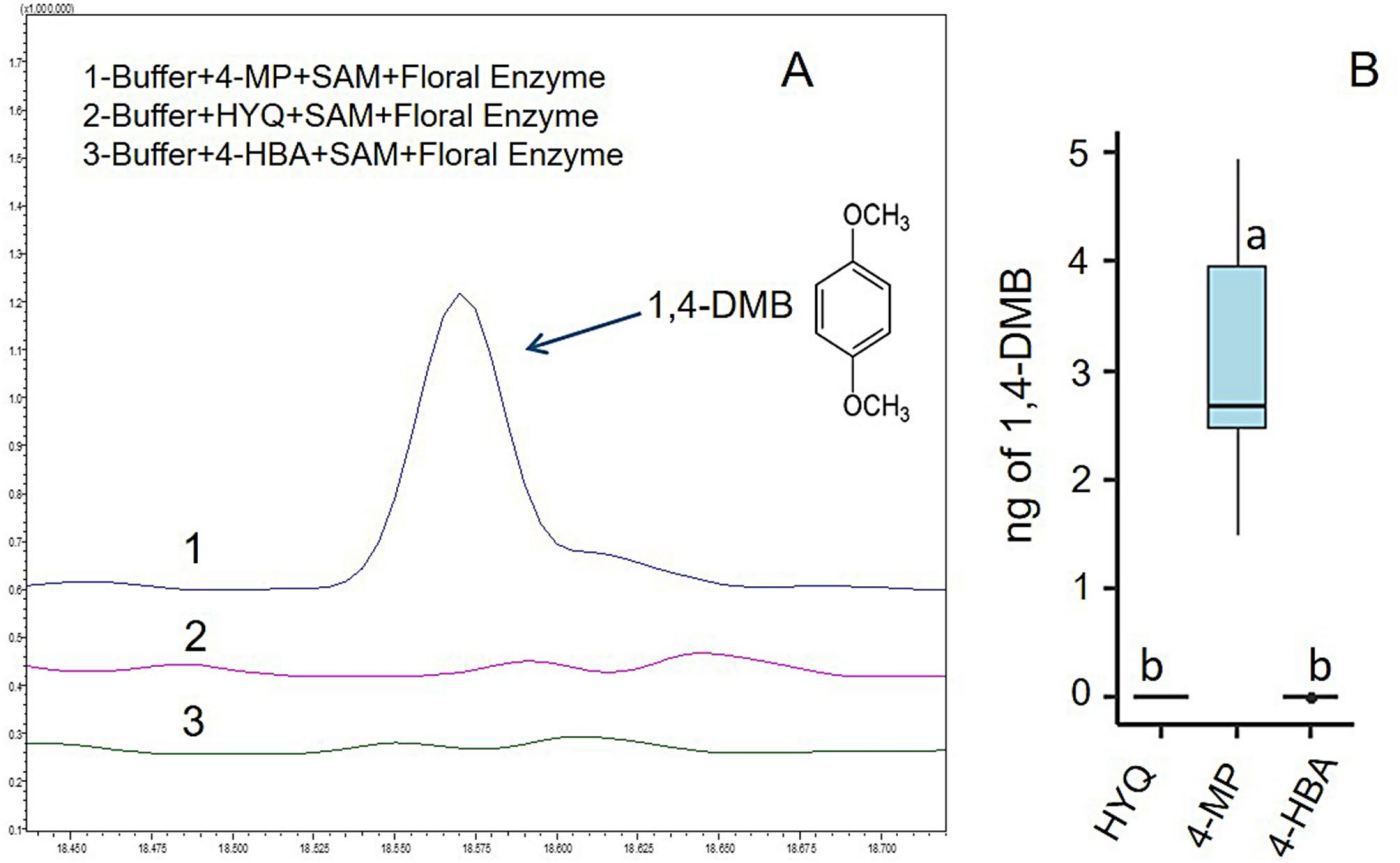
Catalysis of 4-MP to 1,4-DMB. Representative GC chromatograms showing conversion of 4-MP to 1,4-DMB as compared to the substrates HYQ and 4-HBA **(A)**; boxplots showing the amount of 1,4-DMB produced in the reactions **(B)**. Different letters in the box plot indicate significant differences (4-MP, *n* = 7; HYQ, *n* = 5; 4-HBA, *n* = 6; Kruskal-Wallis and Dunn’s tests) at a level of *p* < 0.05

Of the three potential substrates tested (4-HBA, HYQ, 4-MP) on protein extracts of pumpkin petals only the reaction containing 4-MP produced 1,4-DMB (median of 2.67 ng; see Fig. 2A and B), showing that 4-MP is the substrate involved in 1,4-DMB formation in Styrian oil pumpkin flowers.

**Fig. 3:**
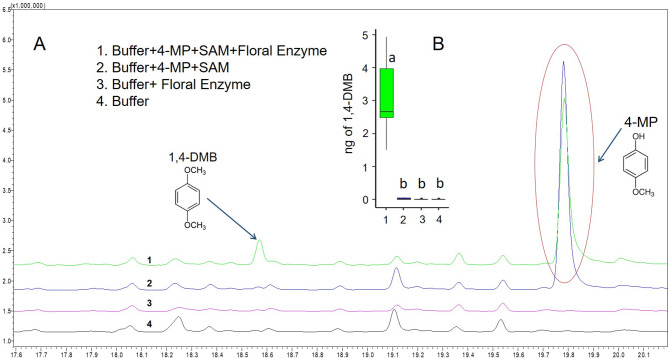
Production of 1,4-DMB from Styrian oil pumpkin floral protein extract. Representative GC chromatograms showing conversion of 4-MP to 1,4-DMB from Styrian oil pumpkin floral OMT crude protein extract as compared to the control samples **(A)**; boxplots showing the amount of 1,4-DMB (in ng) produced in the reactions **(B)**. Different letters in the box plot indicate the significant differences (reaction 1, *n* = 7; reaction 2, *n* = 6; reaction 3, *n* = 4; reaction 4, *n* = 5; Kruskal-Wallis-test and Dunn’s test) at a level of *p* < 0.05

As shown in Fig. 3, only the reaction containing substrate, cofactor, and protein extract was able to catalyse the production of 1,4-DMB. This clearly indicates that crude protein extracts catalyse the formation of 1,4-DMB from 4-MP specifically under the presence of cofactor SAM.

### Identification and phylogenetic analysis of 4MP*O*-methyltransferase from Styrian oil pumpkin

**Fig. 4:**
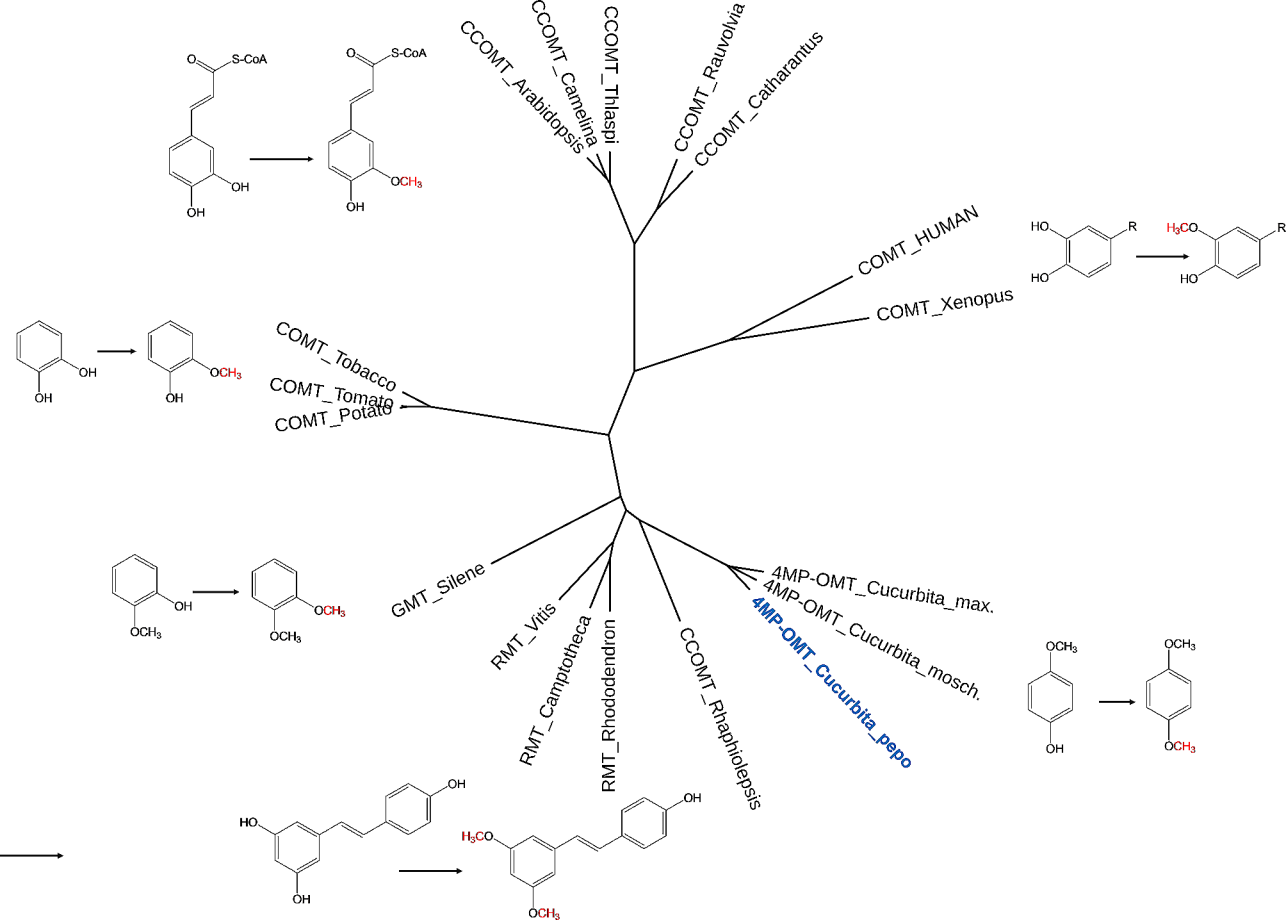
Phylogenetic tree of methyltransferases acting on OH-groups of aromatic ring structures. A prototypic reaction is shown for each branch. The enzymes were annotated as 4-methoxyphenol-*O*-methyltransferase (4MP-OMT); resveratrol-MT (RMT); guaiacol-MT (GMT); catechol-MT (COMT), caffeic-caffeoyl-CoA O-MT (CCOMT); The Accessions: Cp4MP-OMT *Cucurbita pepo* (this paper, highlighted in blue; OR913727); 4MP-OMT *Cucurbita maxima* (XP_022986324); 4MP-OMT *Cucurbita moschata* (XP_022944128.1); *Rhaphiolepis* (LC127201); *Rhododendron* XP_058210860; *Camptotheca* (AWH62807); *Arabidopsis* (AAL07162); *Camelina* (XP_010448306); *Thlaspi* (CAH2077901); *Rauvolfia* (KX687823); *Catharantus* (KAI5664645); human (AAA68927); *Xenopus* (NP_001016357); tobacco (X71430); potato (KAH0753093); tomato (NP_001306101); *Silene* GMT1 (sequence from [42])

By comparing de novo RNA seq data from pumpkin leaves (that do not produce 1,4-DMB; Barman et al., unpublished data) and flowers, we identified a candidate gene for the catalysis of 4-MP to 1,4-DMB. It was strongly upregulated in flower tissue (3272 FPKM) as compared to leaf tissue (10.45 FPKM) and a BLAST-search revealed a homology to *O*-methyltransferases and the potential ability to catalyse the transfer of a methyl-group onto an OH-group of an aromatic ring. We named this sequence *Cp4MP-OMT* and aligned a representative number of homologous methyltransferase sequences using ClustalX. The *Cp4MP-OMT* gene represents a novel branch of methyltransferase sequences clustering together with sequences from other cucurbits (Fig. 4). The encoded enzymes act in 4-position, which is rather rare in plant metabolites. Notably, most other methyltransferases act on aromatic OH-groups in 1,2-position (catechol and derivatives), or in 3 (and 5)-positions as for example in the formation of lignin precursors [51].

**Fig. 5:**
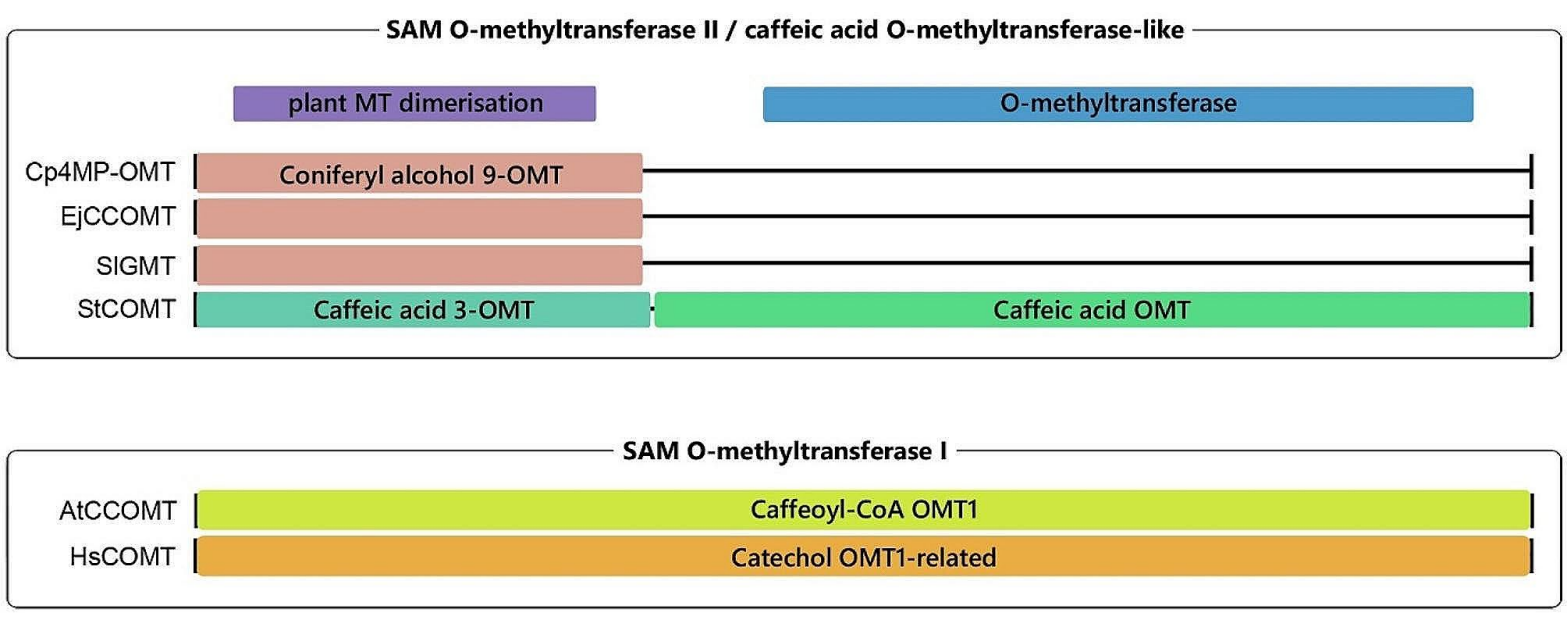
Location of domains and regions in different OMTs. Six protein sequences that are representative for the different branches of the phylogenetic alignment shown in Fig. 4 were analysed using InterPro. The following sequences were used: pumpkin Cp4MP-OMT (this paper), *Rhaphiolepis* EjCCOMT (LC127201), *Silene* SIGMT1 (from [42]), tomato StCOMT (NP_001306101), *Arabidopsis* AtCCOMT (AAL07162), human HsCOMT (AAA68927). The following families, domains and regions were detected and are highlighted in different colours: protein families: SAM-dependent O-methyltransferase class I-type (PROSITE PS51682), SAM-dependent O-methyltransferase class II-type (PROSITE PS51683), caffeic acid O-methyltransferase-like (InterPro IPR016461); domains: plant methyltransferase dimerization domain (IPR012967), O-methyltransferase domain (IPR001077); regions: Coniferyl alcohol 9-OMT (FUNFAM 1.10.10.10:FF:000213), Caffeic acid OMTs (FUNFAM 1.10.10.10:FF:000357 and 3.40.50.150:FF:000061), Caffeoyl-CoA OMT1 homology (FUNFAM 3.40.50.150:FF:000147), Catechol OMT1-related (FUNFAM 3.40.50.150:FF:000054)

As shown by a further investigations of representative protein sequences of OMTs from the phylogenetic analyses of Fig. 4, they group into two families of SAM-dependent OMTs (Fig. 5): SAM-dependent O-methyltransferase class I-type (SAM-OMT I, PROSITE PS51682) and SAM-dependent O-methyltransferase class II-type (SAM-OMT II, PROSITE PS51683). Moreover, the proteins of SAM-OMT II family also group into the family of caffeic acid OMTs (InterPro IPR016461). This family includes Cp4MP-OMT as well as the *Rhaphiolepis*, *Silene* and tomato sequences. These four proteins showed two distinct domains: a plant MT dimerization domain (IPR012967, highlighted in purple in Fig. 5) on the N-terminus that mediates dimerization and an O-methyltransferase domain (IPR001077, highlighted in blue) on the C-terminus that shows transferase activity and utilizes SAM as cofactor. Moreover, a region that is defined as Coniferyl alcohol 9-OMT in the Functional Family database (FUNFAM) was found in *Cucurbita*, *Rhaphiolepis* and *Silene*. The branch of COMTs where the tomato protein was grouped, displays another region: N- as well as C-terminus show homologies to Caffeic acid OMTs (FUNFAM 1.10.10.10:FF:000357 and 3.40.50.150:FF:000061, highlighted in green). The branches including *Arabidopsis* and human OMTs grouped into SAM-OMT I. While AtCCOMT shows a caffeoyl-CoA OMT1 homology (FUNFAM 3.40.50.150:FF:000147, highlighted in yellow), the human sequence revealed a homology to catechol OMT1-related proteins (FUNFAM G3DSA:3.40.50.150:FF:000054, highlighted in orange).

### Expression levels of *Cp4MP-OMT* gene

**Fig. 6:**
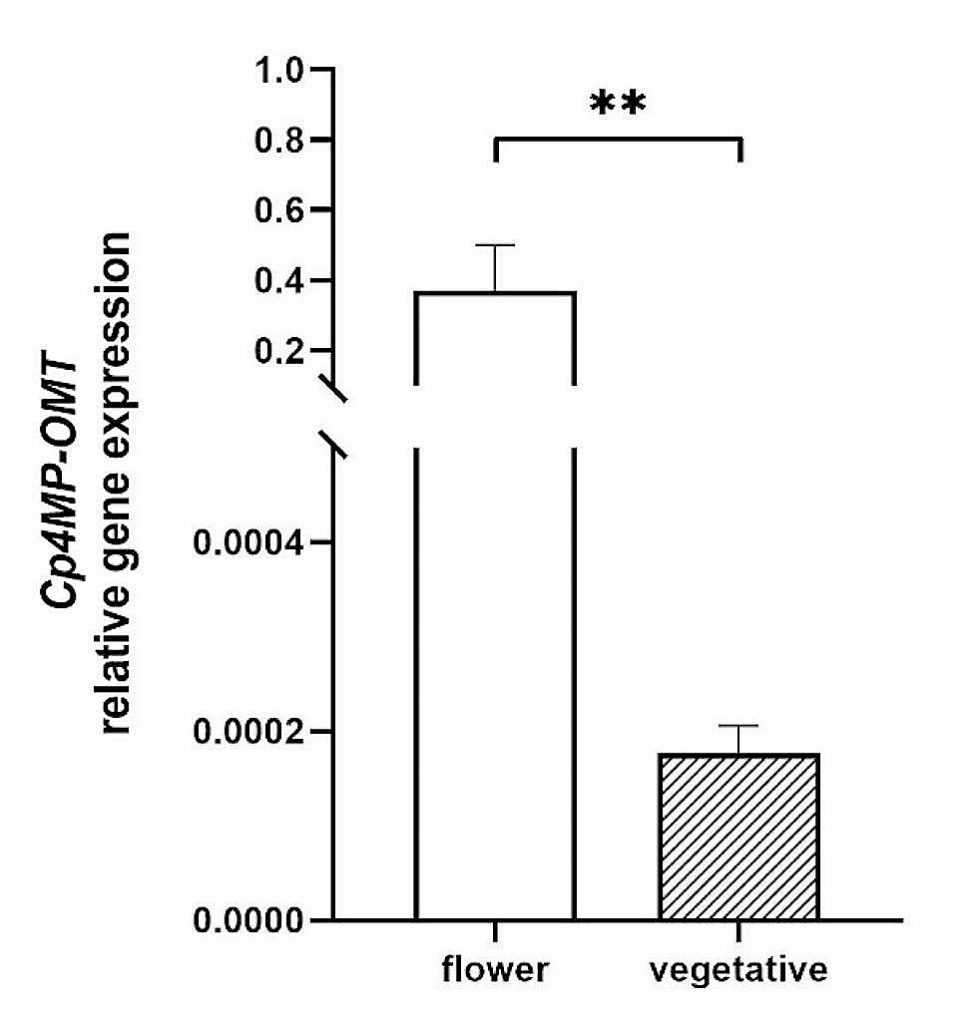
*Cp4MP-OMT* gene expression levels in flower and leaf tissues. Expression levels of *Cp4MP-OMT* (means ± sd) were calculated relative to expression of actin (housekeeping gene), which was set to 1 (*n* = 3). Differences between mean values in flower (1,4-DMB producing) and leaf (non-1,4-DMB producing tendril) tissues are significant (unpaired t-test, asterisks (**) indicate difference at a level of *p* < 0.01)

To verify the difference in gene expression of *Cp4MP-OMT* between flowers and vegetative tissue, as indicated by the transcriptomic data, we compared expression levels of flowers (petal tissue) and vegetative parts of the plant (tendril tissue) using qPCR. Gene expression levels were calculated relative to the housekeeping gene actin, which was set to 1. Figure 6 shows that the expression of *Cp4MP-OMT* in flowers in full bloom (volatile producing tissue) was 0.37 ± 0.13 times that of actin in the same sample. A significantly lower expression of our gene of interest was measured in vegetative tissue of non-volatile producing tendrils: 1.76 × 10^− 4^ ± 3.03 × 10^− 5^ times lower expression of *Cp4MP-OMT* when compared to actin. This means that pumpkin flowers show an about 2100-times higher expression level of *Cp4MP-OMT* compared to vegetative tissue.

### 3.4 Expression and purification of Cp4MP-OMT

**Fig. 7:**
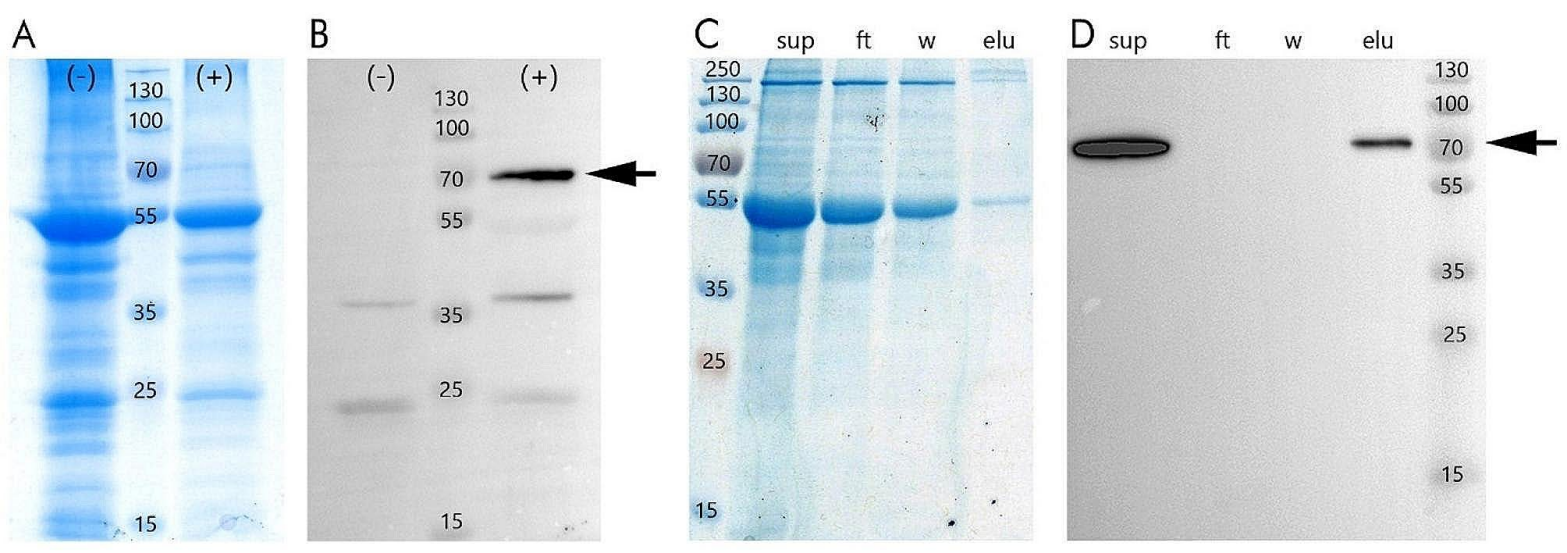
Expression of His-tagged Cp4MP-OMT in *Nicotiana benthamiana*. **A** SDS-PAGE and **B** western blot analyses of crude extract of *Nicotiana benthamiana* leaf tissue with (+) and without (-) recombinantly expressed His-tagged Cp4MP-OMT. Tobacco leaves were transfected with an expression vector either including the sequence for Cp4MP-OMT (+) or without it (-). **C** SDS-PAGE and **D** western blot analyses of His-tagged Cp4MP-OMT purification from crude extract. Lanes are indicated as follows: supernatant (sup), column flow through (ft), wash step (w), and elution (elu). The marker is a molecular mass marker (in kDa). Cp4MP-OMT bands were visualized using an antibody against His(6)-tag (arrows)

*Cp4MP-OMT* was transiently expressed in tobacco leaves to confirm its possible methyltransferase activity. Figure 7A and B show SDS-PAGE and western blot of crude extract from *N. benthamiana* expressing His-tagged Cp4MP-OMT (marked as (+)). Crude extract transiently transformed with an empty expression vector was used as a control (marked as (-)). A protein band of Cp4MP-OMT (arrow) was detected in this crude extract when detected with a monoclonal antibody against His(6)-tag. The extract was used for Ni-TED affinity purification, which is illustrated in Fig. 7C and D. Crude extract as well as purified protein were used for further analyses of OMT activity (compare results Sect. 3.5).

### Assessing the catalysis of 4-MP to 1,4-DMB using recombinant Cp4MP-OMT

**Fig. 8:**
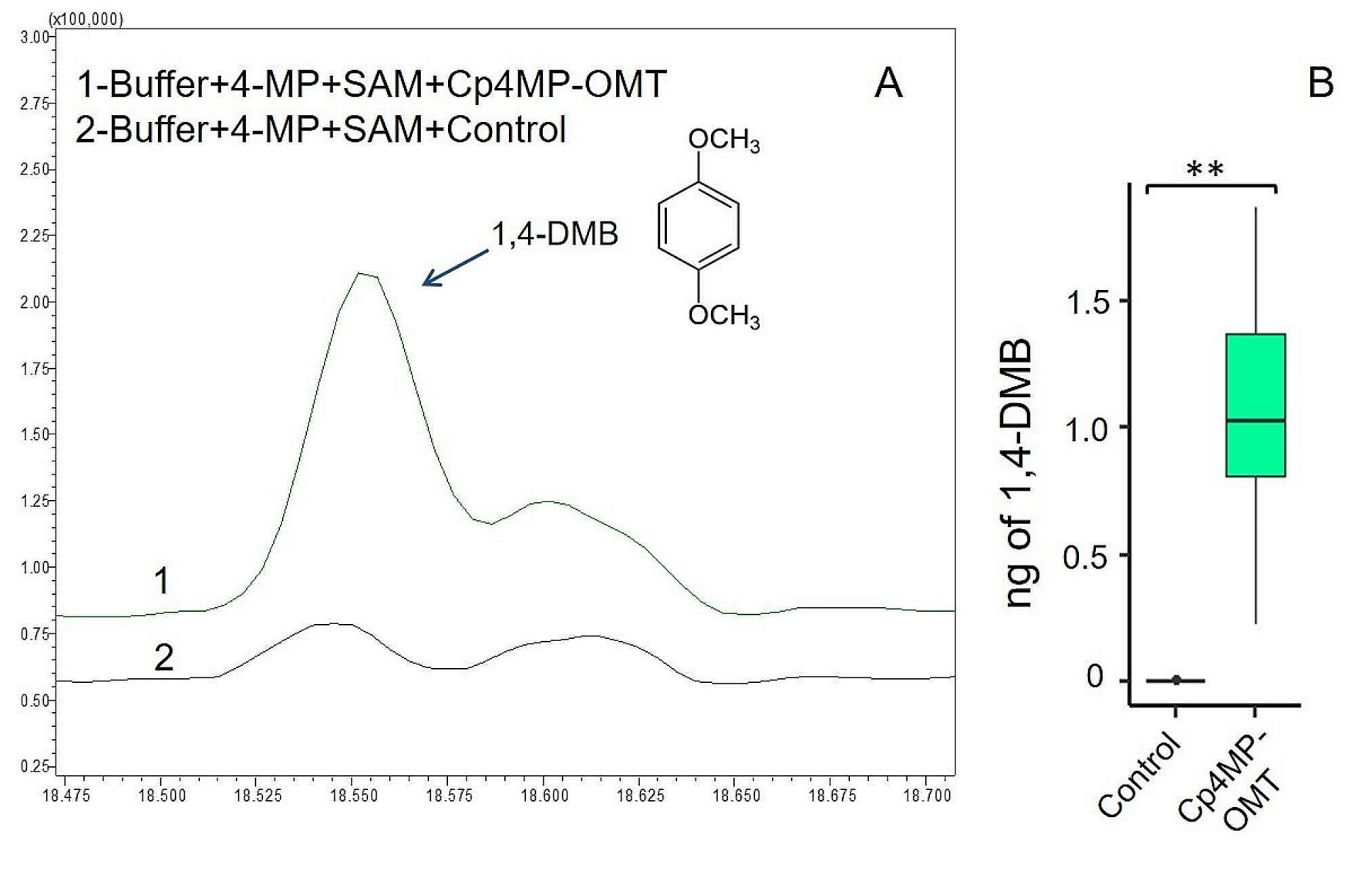
Production of 1,4-DMB from recombinant Cp4MP-OMT. Representative GC chromatograms showing conversion of 4-MP to 1,4-DMB by Cp4MP-OMT as compared to control **(A)**; box plots showing the amount of 1,4-DMB (in ng) produced in the reactions **(B)**. Asterisks (**) in **B** indicate significant difference (Cp4MP-OMT, *n* = 8; control, *n* = 5; Mann-Whitney *U* test) at a level of *p* < 0.005. In **A**, the peak at 18.545 min in 2 is a compound (contaminant) other than 1,4-DMB

As demonstrated by in vitro enzyme assays, 1,4-DMB was formed in the presence of purified recombinant Cp4MP-OMT protein when using the substrate 4-MP as well as the cofactor SAM, while extract from tobacco transformed with an expression vector lacking Cp4MP-OMT (control) did not (Fig. 8). In the headspace samples collected from the reaction mixes, we found 1,4-DMB (median of 1.03 ng) only in recombinant Cp4MP-OMT (Fig. 8).

## Discussion

This study reveals, for the first time, the catalytic activity of protein extracts from *C. pepo* flowers in the formation of 1,4-DMB. Subsequent investigations to discern potential substrates demonstrated that 4-MP is used, and that SAM is needed as a cofactor for this reaction. In contrast, neither 4HBA nor HYQ yielded measurable 1,4-DMB production. In the pursuit of elucidating the responsible protein for this observed OMT activity, we analysed transcriptomic datasets from *C. pepo* and found a putative OMT sequence that was significantly upregulated in 1,4-DMB-producing tissue of flowers compared to non-1,4-DMB-producing leaf tissue. Using this nucleotide sequence to express and purify recombinant Cp4MP-OMT, we proved its functionality in an enzymatic assay: Cp4MP-OMT catalysed 1,4-DMB by using 4-MP and SAM. Furthermore, we demonstrated by qPCR that *Cp4MP-OMT* is 2100 times stronger expressed in flowers than leaf tissues, which confirmed the findings from the transcriptomic datasets.

Our finding that the crude floral OMT extract catalyses the formation of 1,4-DMB from the substrate 4-MP (see Figs. 2 and 3) goes along with a previous finding that reports the formation of its structurally isomeric compound 1,2-DMB from 2-MP in *Silene latifolia* flowers using crude OMT extract [42]. In *S. latifolia* as well as in other plants [17, 52], flowers emit both the substrate 2-MP and 1,2-DMB. On the contrary, some flowering species release high amounts of 1,2-DMB but not 2-MP [9, 21], which indicates that 2-MP is rapidly catalysed to 1,2-DMB by OMTs in these species. Similarly, in the floral headspace of Styrian oil pumpkin, we detected high amounts of 1,4-DMB, but did not find any traces of the substrate 4-MP (Barman et al. unpublished). This is also true for other plants that release 1,4-DMB but not 4-MP (e.g., *Catasetum* [23], *Lithophragma* [21], *Cucurbita* spp [25]. Generally, 4-MP is emitted from a few plant species [53, 54], out of which one emits 4-MP together with 1,4-DMB (from the inner bark of *Tabebuia impetiginosa* [54]. This suggests that 4-MP is a potential substrate for 1,4-DMB formation in various plants but is rapidly catalysed to 1,4-DMB by OMTs in most plant species leaving no traces of 4-MP in their scent profile. In the future, it would be interesting to investigate the biosynthetic pathway enzymes and substrates involved in the formation of 4-MP.

Examination of protein sequences of some of the OMTs from the phylogenetic tree using InterPro revealed that all of them classified either into class I or class II of SAM-dependent OMTs (see Fig. 5). This indicates that all of them use SAM as a cofactor in their enzymatic reactions. Delineation of these classes is based on structural attributes of their catalytic domains: SAM-OMTs of class I feature a Rossmann-like alpha/beta structure, while SAM-OMTs of class II show a TIM beta/alpha-barrel structure [55]. Moreover, the plant OMTs depicted in Fig. 5 were identified as caffeic acid OMT-like, indicating a potential substrate affinity for caffeic acid. They also show a distinctive N-terminal plant-specific methyltransferase dimerization known for mediating dimerization of these molecules. In addition, an OMT domain is found on the C-terminus that is responsible for the utilization of the cofactor SAM.

Phylogenetic analyses using different known OMTs (compare Fig. 4) outline the divergence of catechol OMTs from animals and solanaceous plants. Enzymes involved in lignin precursor formation and those *O*-methylating the bulky stilbene resveratrol [56] occupy separate branches. Though several OMTs can methylate more than one substrate [57, 58], the main principal reaction is likely maintained within each branch. The alignment underscores a profound impact of the primary sequence of OMTs on their use of aromatic substrates with substrate specificity for *O*-methylation of hydroxyl groups on aromatic rings favouring the ortho (1,2-) dihydroxy (methoxy) configuration. The *O*-methylation of substrates in para (1,4-) position, as demonstrated here for pumpkin Cp4MP-OMT and 1,4-DMB, is infrequent but has also been reported for CCOMT enzyme from *Rauvolvia* [58]. Other plant OMTs have been reported to form 2-MP (catechol-*O*-methyltransferase, see [59]) or 1,2-DMB (guaiacol-*O*-methyltransferase, see [42]) with a methyl group getting transferred to a distinct hydroxy group at ortho/meta/para-position [39].

Our recombinant methyltransferase Cp4MP-OMT added to the rather short list of proteins with proven methyltransferase activity, whereas hundreds of MT sequences are available in public databases. This raised the question, whether sequence similarity itself is a good predictor of substrate specificity. Using the sequence from *C. pepo* as query, we found two sequences from plants (*Salix* [60] and *Fragaria* [24]), which are known to produce 1,4-DMB. When integrated in the phylogenetic tree of Fig. 4, both sequences map to the contact zone between *Cucurbita* sequences and the resveratrol branch (data not shown). This suggests that the branch of Cucurbitaceae has evolved an “own” version of OMTs specific for 1,4-methylation.

As described in the literature, the *O*-methyltransferase of *Vitis vinifera* acting on resveratrol has a dual function and is acting twice on its substrate in a sequential process [59]. Surprisingly, Cp4MP-OMT only catalyses the methylation of 4-methoxyphenol in the final methylation step to 1,4-DMB, but not the preceding methylation of Hydroquinone (4-hydroxyphenol) to 4-MP (see Fig. S1B). This, as well as the findings from the phylogenetic alignment described above, suggest the presence of a second methyltransferase that has not been identified yet. It must be mentioned that the relative activity of first vs. second methylation reaction of *V. vinifera* resveratrol-OMT [59] can be modified significantly by exchanging only a few amino acids. Moreover, these exchanges also shift the preference from 3-methyl resveratrol to 3/5-dimethyl resveratrol. Keeping this in mind, the great diversities in substrate specificity as well as positioning of *O*-methylation of MTs could be explained.

## Conclusion

This study presents the discovery of the novel *O*-methyltransferase Cp4MP-OMT from *Cucurbita pepo* along with its associated substrate. Gene expression of *Cp4MP-OMT* is highly upregulated in the volatile-emitting tissue of flowers. Through experimentation, we have substantiated its capacity to catalyse the final methylation step in the biosynthesis of the pivotal floral scent attractant, 1,4-DMB, utilizing 4-MP as the substrate. Considering the importance of 1,4-DMB in attracting both pollinators and florivores to flowers [8, 30] enhanced comprehension of this enzyme and its substrate holds promise for ecological insights and advancements in plant breeding endeavours.

### Electronic supplementary material

Below is the link to the electronic supplementary material.

Supplementary Material 1

Supplementary Material 2

## Data Availability

The sequence for Cp4MP-OMT is available in GenBank under the accession number OR913727. Other data are available from corresponding author on request.
