# Supplementary material for: A novel O-methyltransferase Cp4MP-OMT catalyses the final step in the biosynthesis of the volatile 1,4-dimethoxybenzene in pumpkin (Cucurbita pepo) flowers
Source: BMC Plant Biol. 2024 Apr 17;24:294. doi: 10.1186/s12870-024-04955-3 (PMC11022444; doi:10.1186/s12870-024-04955-3)
Supplement: Supplementary file 1 — Supplementary Material 1 [file 12870_2024_4955_MOESM1_ESM.pdf]

Supplementary file

A novel *O*-methyltransferase Cp4MP-OMT catalyses the final step in the biosynthesis of the volatile 1,4-dimethoxybenzene in pumpkin (*Cucurbita pepo*) flowers

Marion Christine Hoepflinger[1,†], Monica Barman[1, 2†], Stefan Doetterl[1], Raimund Tenhaken[1*]

[†]Marion Christine Hoepflinger and Monica Barman contributed equally to this work.

[*]Correspondence: Raimund Tenhaken

[1]Department of Environment & Biodiversity, Paris Lodron University Salzburg, Hellbrunnerstrasse 34, 5020 Salzburg, Austria.

[2]Leibniz Institute of Vegetable and Ornamental Crops (IGZ), Theodor-Echtermeyer-Weg 1, 14979, Großbeeren, Germany.

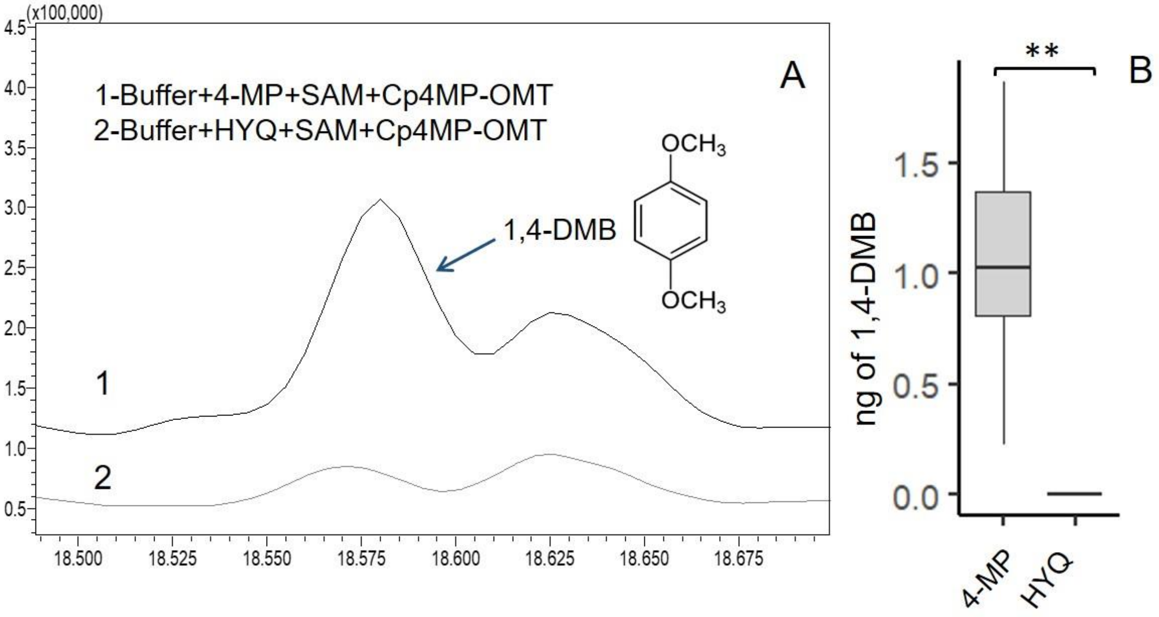

Figure S1 Production of 1,4-DMB by Cp4MP-OMT from 4-MP as compared to HYQ.

Representative GC chromatograms showing formation of 1,4-DMB from 4-MP by Cp4MP-OMT as compared to substrate HYQ (A); box plots showing the amount of 1,4-DMB (in ng) produced in the reactions (B). Asterisks (**) in B indicate significant difference (4-MP, $n = 8$; HYQ, $n = 4$; Mann-Whitney U test) at a level of $p < 0.01$. In A, the peak at 18.56 min in 2 is a base contaminant compound other than 1,4-DMB.
