# Supplementary material for: A novel O-methyltransferase Cp4MP-OMT catalyses the final step in the biosynthesis of the volatile 1,4-dimethoxybenzene in pumpkin (Cucurbita pepo) flowers
Source: BMC Plant Biol. 2024 Apr 17;24:294. doi: 10.1186/s12870-024-04955-3 (PMC11022444; doi:10.1186/s12870-024-04955-3)
Supplement: Supplementary file 2 — Supplementary Material 2 [file 12870_2024_4955_MOESM2_ESM.pdf]

Supplementary File 2:

A novel *O*-methyltransferase Cp4MP-OMT catalyses the final step in the biosynthesis of the volatile 1,4-dimethoxybenzene in pumpkin (*Cucurbita pepo*) flowers

Marion Christine Hoepflinger[1,†], Monica Barman[1, 2†], Stefan Doetterl[1], Raimund Tenhaken[1*]

[†]Marion Christine Hoepflinger and Monica Barman contributed equally to this work.

[*]Correspondence: Raimund Tenhaken

[1]Department of Environment & Biodiversity, Paris Lodron University Salzburg, Hellbrunnerstrasse 34, 5020 Salzburg, Austria.

[2]Leibniz Institute of Vegetable and Ornamental Crops (IGZ), Theodor-Echtermeyer-Weg 1, 14979, Großbeeren, Germany.

Supplementary File 2:

Uncropped and not overlayed images of SDS-PAGE gels and PVDF membranes used in the western blot analyses of Figure 7: Expression of His-tagged Cp4MP-OMT in *Nicotiana benthamiana*.

Supplementary File 2: Uncropped and not overlayed images of SDS-PAGE gels and PVDF membranes used in western blot analyses of Figure 7: Expression of His-tagged Cp4MP-OMT in *Nicotiana benthamiana*.

(a) SDS-PAGE and **(b)** western blot analyses of crude extract of *Nicotiana benthamiana* leaf tissue with (+) and without (-) recombinantly expressed His-tagged Cp4MP-OMT. Tobacco leaves were transfected with an expression vector either including the sequence for Cp4MP-OMT (+) or without it (-). **(c)** SDS-PAGE and **(d)** western blot analyses of His-tagged Cp4MP-OMT purification from crude extract. Lanes are indicated as follows: supernatant (sup), column flow through (ft), wash step (w), and elution (elu). The marker is a molecular mass marker (in kDa). Cp4MP-OMT bands were visualized using an antibody against His(6)-tag (arrows).

<u>SDS-PAGE gel of **Figure 7a:**</u>

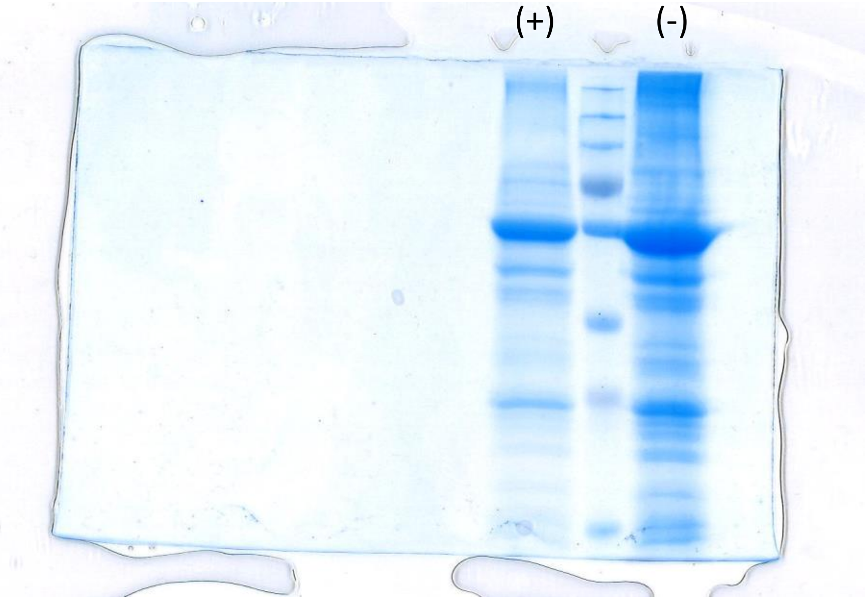

<u>PVDF-membrane of the western blot shown in **Figure 7b:**</u>
Picture of marker bands detection **in visible light**.

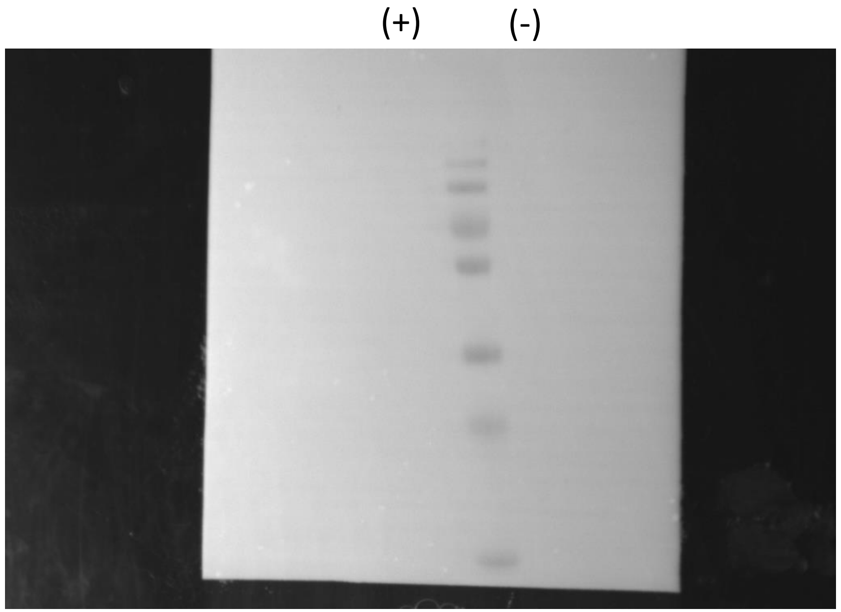

<u>PVDF-membrane of western blot shown in **Figure 7b:**</u>
His-tag detection of the membrane shown on the left using **chemiluminescence**.

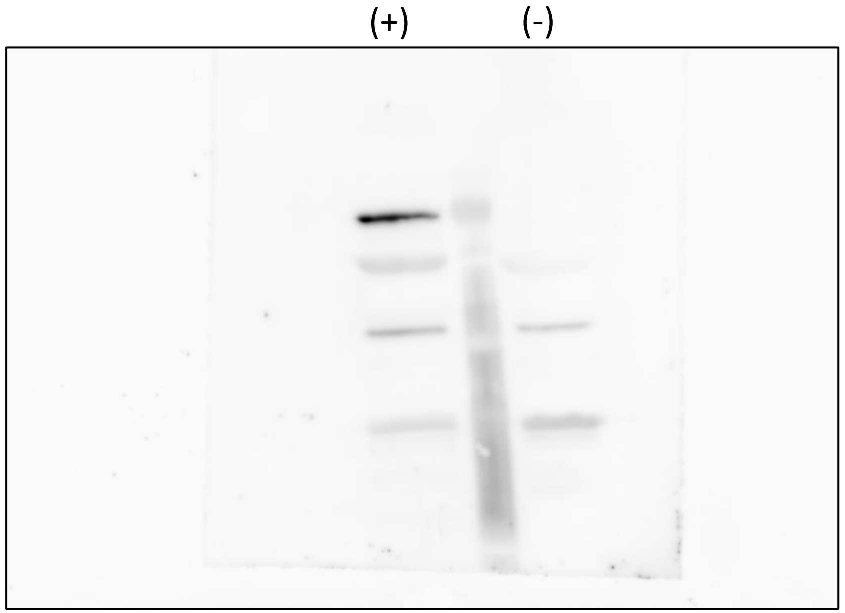

Uncropped image of SDS-PAGE gel of Figure 7:

(c) SDS-PAGE and **(d)** western blot analyses of His-tagged Cp4MP-OMT purification from crude extract. Lanes are indicated as follows: supernatant (sup), column flow through (ft), wash step (w), and elution (elu). The marker is a molecular mass marker (in kDa). Cp4MP-OMT bands were visualized using an antibody against His(6)-tag (arrows).

SDS-PAGE gel of **Figure 7c:**

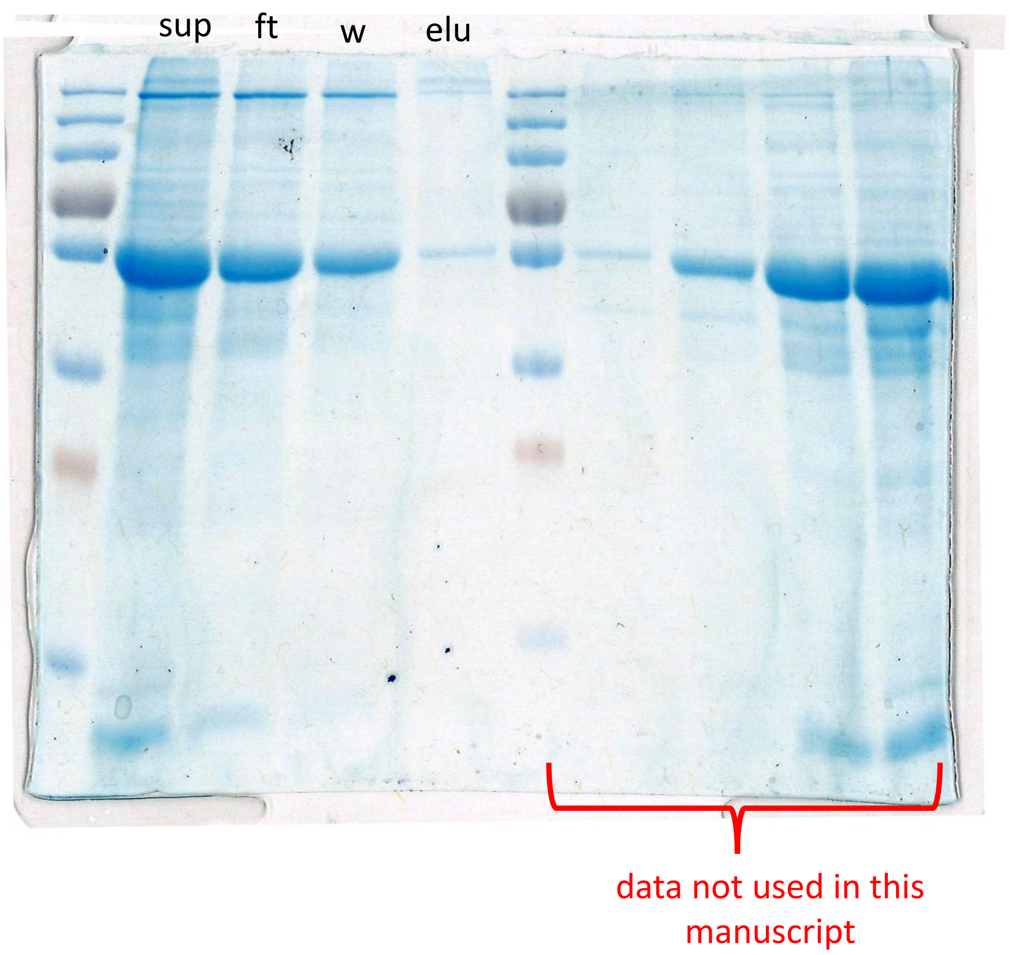

Uncropped and not overlayed images PVDF membranes used in western blot analyses of Figure 7: Expression of His-tagged Cp4MP-OMT in *Nicotiana benthamiana*.

(d) western blot analyses of His-tagged Cp4MP-OMT purification from crude extract. Lanes are indicated as follows: supernatant (sup), column flow through (ft), wash step (w), and elution (elu). The marker is a molecular mass marker (in kDa). Cp4MP-OMT bands were visualized using an antibody against His(6)-tag (arrows).

<u>PVDF-membrane of the western blot shown in **Figure 7d:**</u>
Picture of marker bands detection **in visible light**.

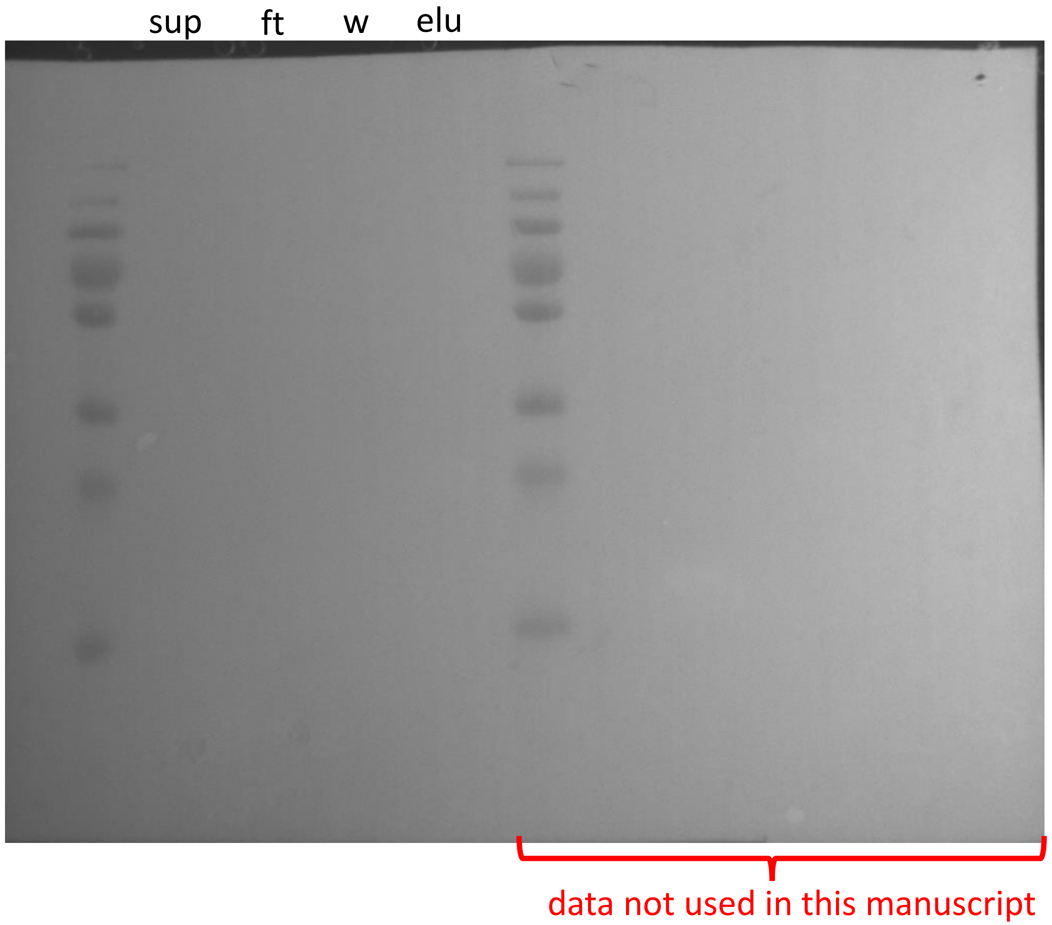

<u>PVDF-membrane of western blot shown in **Figure 7d:**</u>
His-tag detection of the membrane shown on the left using **chemiluminescence**.

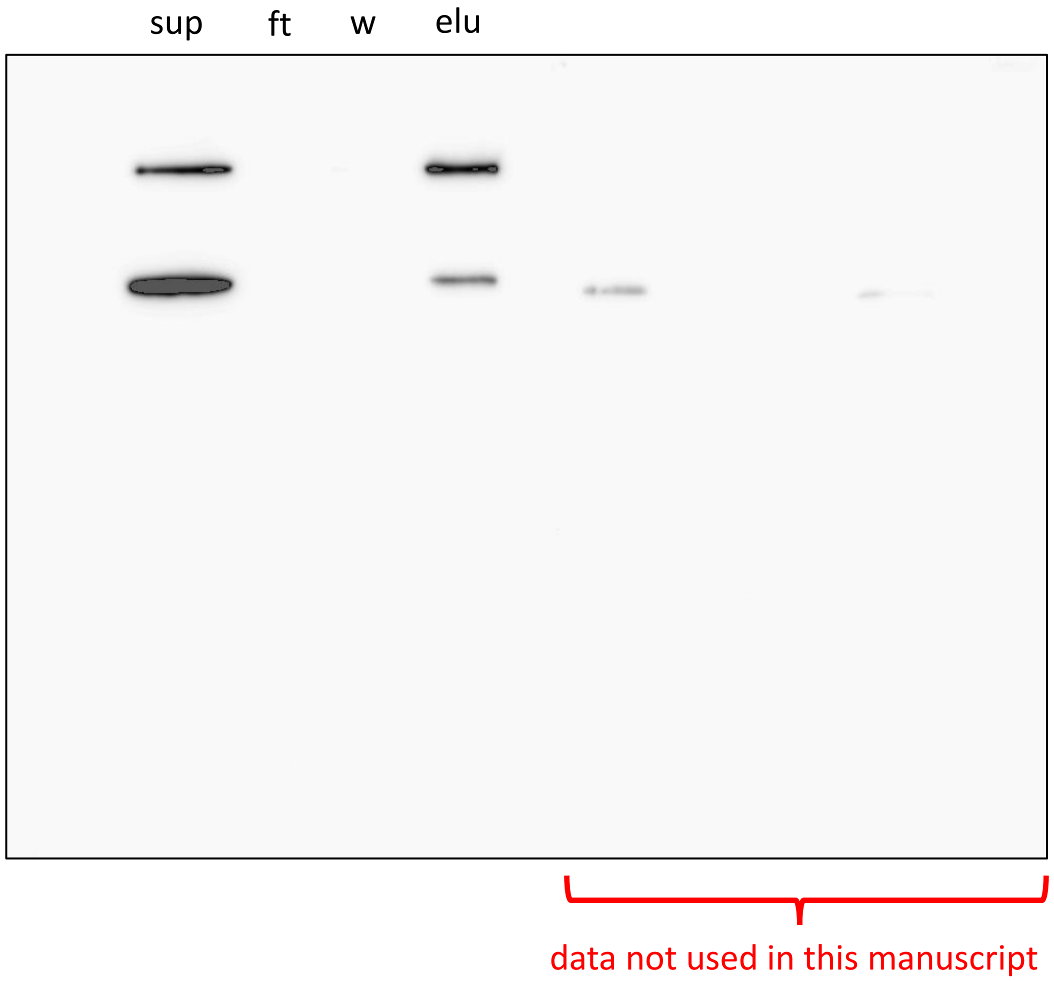

As anticipated, the His-tagged Cp4MP-OMT exhibited a distinct band around 70 kDa, as evident from the PVDF membrane chemiluminescence detection image. Additionally, there was another band observed at approximately 250 kDa, indicating a multimeric form of His-tagged Cp4MP-OMT. To enhance clarity, this particular band was omitted from the main manuscript.
